# Kinestesia: the prefrontal EEG response to a cognitive-motor intervention is graded by baseline cognitive status

**DOI:** 10.3389/fnagi.2026.1931028

**Published:** 2026-09-22

**Authors:** Jorge F. Bosch-Bayard, Judith Guerrero-Sauzameda, Laura Navarrete-Torres, Liane Aguilar-Fabre, Rene F. Rodriguez-Valdes, Rodolfo I. Bosch-Bayard, Andrei Martinez-Finkelstein, Gracia Castro-Luna, Giuseppe A. Chiarenza

**Affiliations:** 1 Carl von Ossietzky Universität Oldenburg, Faculty of Psychology, Oldenburg, Germany; 2 Clínica del Sistema Nervioso, Universidad Autónoma de Querétaro, Querétaro, Mexico; 3 Centro Integrador del Movimiento, Mente y Conducta, Querétaro, Mexico; 4 Vithas Málaga, Grupo Hospitalario Vithas, Málaga, Spain; 5 State University of Zanzibar, Zanzibar, Tanzania; 6 Universidad de Ciencias Médicas de La Habana, Havana, Cuba; 7 Universidad de Almería, Facultad de Medicina, Almería, Spain; 8 Centro Internazionale Disturbi di Apprendimento, Attenzione, Iperattività, Milan, Italy

**Keywords:** cognitive reserve, HarMNqEEG, kinestesia, normative database, quantitative EEG, sensory-motor cognitive intervention, spectral biomarkers, state-dependent neuroplasticity

## Abstract

**Background:**

This is a companion neurophysiological study (*N* = 19) to a recently published exploratory pilot of the Kinestesia intervention — combining therapeutic videogames, rhythmic gait, and sensory stimulation — in older adults spanning the cognitively healthy (HC, *n* = 10) to mild cognitive impairment (MCI, *n* = 9) spectrum. That paper, published in Archives of Gerontology and Geriatrics, reported BH-corrected behavioural gains; here we ask whether the resting-state electrocortical response is graded by baseline cognitive status.

**Methods:**

Resting-state EEG was recorded before and after 30 sessions in 19 participants (9 MCI, MoCA < 26; 10 HC, MoCA 26–29). Age-referenced Z-scores were computed for eleven pre-specified clinical EEG indices using the Harmonized Functional EEG Derived Phenotypes normative pipeline (HarFEDPNorms). The primary analysis treated the 19 participants as a single sample and correlated baseline MoCA with the pre-to-post change in each index (Spearman ρ), with Benjamini-Hochberg (BH) FDR correction. We assessed robustness to educational attainment using partial correlation.

**Results:**

Eight of the eleven pre-specified indices showed a BH-corrected continuous association with baseline MoCA. The strongest were the Engagement Index (EI) and the Alpha/Beta Ratio (ABR) at Fp2 (EI: ρ = +0.704, *q* = 0.008; ABR: ρ = −0.687, *q* = 0.008): higher baseline cognitive status was associated with greater prefrontal activation after the intervention, and lower status with the reverse. MoCA and education were substantially correlated at the sample level (*r* = 0.596; 36% shared variance, driven by the HC/MCI contrast), and the primary prefrontal effect (EI Fp2) remained significant after adjusting for education (partial ρ = 0.50, *p* = 0.033).

**Conclusion:**

In this small, uncontrolled sample, the electrocortical response to Kinestesia was graded by baseline cognitive status, with a robust prefrontal engagement effect (EI and ABR at Fp2; BH *q* < 0.01). Findings are exploratory and hypothesis-generating, pending a randomised controlled trial. The HarFEDPNorms pipeline offers a device-independent framework for individualised EEG tracking in intervention research.

## 1 Introduction

Kinestesia was first presented in Bosch-Bayard et al. (2026a), a single-arm exploratory pilot study (*N* = 20 for behavioral outcomes, *N* = 19 with valid EEG) in which the intervention—combining therapeutic videogames, rhythmic gait, and sensory stimulation—produced BH-corrected improvements in verbal memory and functional capacity across a continuous MoCA range of 14–29. The present paper is the companion neurophysiological report: it asks what changes in the resting-state electrocortical spectrum accompany those behavioral gains, and whether the pattern depends on baseline cognitive status.

Mild cognitive impairment sits at a critical inflection point in the aging brain. The characteristic MCI spectral profile—excess delta and theta power over temporal and central regions, slowing of the dominant occipital alpha rhythm, and disruption of frontoparietal coherence—has been replicated across dozens of studies and is now considered a reliable functional biomarker (Jeong, 2004; van der Hiele et al., 2007). Critically, this profile reflects a dynamic network state rather than a fixed structural lesion: it fluctuates with arousal, pharmacological challenge, and cognitive load, and partially normalizes in response to successful cognitive interventions (Klimesch, 1999). Whether structured cognitive-motor training can shift this profile in a clinically meaningful direction—and whether the direction depends on the individual's baseline neurophysiological state—remains an open question that motivates the present study.

The neurophysiological rationale for sensory-motor cognitive interventions in MCI, a population in whom sedentary behavior is itself a modifiable risk factor for cognitive decline (Cai et al., 2023), rests on convergent evidence from exercise neuroscience and systems neuroscience. Aerobic exercise acutely modulates cortical spectral dynamics in older adults through at least two mechanisms: noradrenergic and cholinergic arousal, which shift the spectral balance toward faster frequencies (Hillman et al., 2008), and hippocampal theta entrainment, driven by locomotor rhythm and supporting working memory encoding and consolidation (Buzsáki, 2002; Erickson et al., 2011). When cognitive load is superimposed on motor activity—as in dual-task paradigms, exergames, or structured game-based programmes—additional prefrontal executive networks are recruited, potentially amplifying and sustaining these acute spectral shifts into longer-term neuroplastic change (Bherer et al., 2013; Lambourne and Tomporowski, 2010).

A central empirical question is whether the electrocortical response to sensory-motor cognitive interventions is graded by the participant's baseline cognitive status. In the exercise-EEG literature, resting-state spectral profiles before an intervention predict the direction and magnitude of post-exercise change (Crabbe and Dishman, 2004), and analogous heterogeneity has been described in combined cognitive-motor programmes (Bherer et al., 2013). Whether a similar gradient operates in MCI populations—where baseline electrocortical profiles differ systematically from those of cognitively healthy adults (Jeong, 2004; van der Hiele et al., 2007)—is the exploratory question we address here.

Of the N=22 participants who completed the full intervention, 19 had valid pre- and post-intervention resting-state EEG recordings: 9 MCI (MoCA < 26, mean age 69.8 ± 3.7 years) and 10 HC (MoCA ≥26, mean age 69.5 ± 3.5 years). One HC participant was excluded due to excessive post-intervention artifacts. We retained individual MoCA scores (range 14–29) as a continuous cognitive reserve index (Khalaila et al., 2024; Liu et al., 2024; Nelson et al., 2021). For full demographic details, see Table 1. Information can also be found in Bosch-Bayard et al. (2026a).

**Table 1:** Participant characteristics.

Characteristic	MCI (*n* = 9)	HC (*n* = 10)
Age, years (mean ± SD)	69.8 ± 3.7	69.5 ± 3.5
Female sex, *n* (%)	6 (67%)	7 (70%)
Education, years (mean ± SD)	7.3 ± 2.5	13.9 ± 2.6
BMI (mean ± SD)	29.0 ± 2.9	25.7 ± 4.0
MoCA total score (mean ± SD)	21.6 ± 3.8	27.4 ± 1.3

Values are mean ± SD unless stated otherwise. BMI, body mass index; MoCA, montreal cognitive assessment.

We used the HarFEDPNorms pipelines (Bosch-Bayard et al., 2001, 2020a, 2026b) to compute age-referenced Z-scores for a set of pre-specified clinical EEG indices. We hypothesized, as an exploratory question, that across the 19 participants the pre-to-post change in these indices would follow a continuous gradient along baseline MoCA—greater normalization of prefrontal activation indices in participants with higher baseline cognitive status. Given the sample size, full-spectrum and band-level analyses were treated as descriptive only and are not reported here.

## 2 Materials and methods

### 2.1 Participants and study design

This companion EEG study to Bosch-Bayard et al. (2026a) uses the same participants, setting, and intervention. More details of the study design, inclusion criteria, and ethics approval are reported there. All procedures complied with the Declaration of Helsinki; ethics approval was granted by the Ethics Committee of the Faculty of Medicine, Universidad Autónoma de Querétaro (protocols 02-03/11/2016 and 03-01/12/2016; institutional grant FOPER No. 143834); written informed consent was obtained.

Briefly, N=22 older adults (≥60 years) were recruited from the Plan Vida center, in Queretaro, Mexico, following non-probabilistic purposive sampling from an initial screen of 70 individuals. Eligibility required independent ambulation, absence of uncontrolled systemic or neurodegenerative disease, no clinically significant depression (Yesavage GDS < 5), and attainment of the Six-Min Walk Test functional threshold. Of these, 19 participants had complete pre- and post-intervention EEG recordings and constitute the analysis sample: 9 MCI (MoCA < 26) and 10 HC (MoCA ≥ 26). Individual MoCA scores were retained as a continuous covariate in all analyses. Table 1 presents participant characteristics.

### 2.2 The Kinestesia intervention

Participants completed 30 sessions (3 × /week, ~10 weeks, 60 min/session) at the Plan Vida center, in Queretaro, Mexico, supervised by trained physiotherapists. Each session comprised alternating active play and rest blocks (~40 min active), with games sequenced by sensory-motor cognitive complexity. An adaptive difficulty algorithm operated both within and across sessions, adjusting stimulus speed, target density, and response windows in real time based on performance trajectories. Twenty-three games spanning five functional categories (gait sequencing, visual attention, perceptual categorization, abstraction, and arithmetic) were included. All session data were automatically logged.

### 2.3 EEG recording and pre-processing

Resting-state EEG was recorded within two weeks before the first Kinestesia session (Pre) and within two weeks after the final session (Post). Participants sat comfortably with eyes closed in a quiet, dimly lit room. Recordings used the standard 10–20 system with 19 active electrodes (Fp1, Fp2, F3, F4, C3, C4, P3, P4, O1, O2, F7, F8, T3, T4, T5, T6, Fz, Cz, Pz), linked ears reference, and electrode impedances below 10 kΩ. Acquisition used a Medicid-V system (Neuronic) at a 200 Hz sampling rate with a hardware bandpass of 0.5–70 Hz.

Pre-processing was performed in MATLAB. Continuous recordings were segmented into non-overlapping epochs; segments contaminated by ocular, muscular, cardiac, movement, or electrode artifacts were identified by visual inspection and rejected by two experienced neurophysiologists, blind to group and time-point, with disagreements resolved by consensus; a minimum of 2 min of artifact-free EEG was retained per recording. Power spectral density was estimated via Fast Fourier Transform (FFT) at 0.39 Hz resolution across 0.78–19.15 Hz (48 frequency bins). Data were re-referenced to the average reference montage and rescaled by the global scale factor (GSF) (Hernández et al., 1994) to reduce inter-individual variance arising from skull thickness, tissue conductivity, and other factors not related to neurophysiology.

### 2.4 Normative pipeline and Z-score computation

Raw spectral estimates were submitted to the HarFEDPNorms pipeline (Bosch-Bayard et al., 2026b) to obtain age-dependent Z-scores for the model variables. Z=0 represents the mean of the age-matched healthy-adult reference population; a post-intervention shift toward *Z* = 0 in a participant whose pre-intervention Z was elevated constitutes partial normalization. Z-scores were computed for seven primary clinical EEG indices with established interpretive frameworks as described in Bosch-Bayard et al. (2026b). Table 2 summarizes the primary clinical indices used here.

**Table 2:** Primary clinical EEG indices.

Index	Formula	General interpretation in literature
EI^*^	β/(α+θ) (engagement index)	Higher values: increased cortical arousal and attentional engagement
ABR^*^	α/β Ratio	Higher values: relaxed, disengaged states; inverse of EI
TBR^*^	θ/β Ratio	Elevated: inattention and cognitive hypoarousal; established MCI marker
TAR^*^	θ/α Ratio	Elevated: relative spectral slowing; associated with cognitive fatigue and MCI
Valence	α(F3)/β(F3) – α(F4)/β(F4)	Frontal alpha asymmetry; positive: approach motivation
Arousal	β(F3 + F4)/[δ(F3 + F4) + θ(F3 + F4)]	Frontocentral Composite activation index (F3, F4)
TBHR^*^	θ/β_high_ (theta/beta-high ratio)	Higher values: slow-wave excess relative to high-beta; sensitive to frontotemporal theta/beta imbalance

^*^These indices are calculated one for each electrode. Valence and Arousal are just one scalar; therefore, the total number of indices is, in general, number_of_electrodes_ ^*^ 5 + 2. In our case, 97.

Z-probabilistic scores were calculated for these indices, according to the normative procedures introduced in Bosch-Bayard et al. (2026b) (HarFEDPNorms), where norms were calculated based on the HarMNqEEG normative database (Li et al., 2022) with 1,564 subjects from nine countries and 19 sites.

### 2.5 Statistical analysis

All analyses used Python 3.11 (SciPy 1.11). The primary analysis treated the 19 participants as a single sample. For each clinical index and electrode, we computed the Spearman rank correlation across the 19 participants between baseline MoCA and the pre-to-post change in the Z-score, ΔZ = Z(Post)–Z(Pre); this is what we refer to as the “MoCA gradient”. It is a subject-wise correlation (one value per participant), not a spatial gradient across electrodes; the scalp maps display the electrode-wise correlation coefficients. For example, for EI at Fp2 the gradient is the Spearman ρ between each participant's baseline MoCA and their ΔEI(Fp2). Significance was assessed by permutation, and *p*-values were FDR-controlled across the eight (97) pre-specified indices using the Benjamini–Hochberg (BH) procedure (*q* < 0.05). Effect sizes are Cohen's d (within-group change) and the correlation coefficient ρ (gradient); with *n* = 19, large ρ values can be influenced by individual observations and are interpreted with caution (Cohen, 1988; Lakens, 2013). To assess whether the gradient was confounded by educational attainment, we recomputed the primary correlations as partial Spearman correlations controlling for years of education. Indices not surviving FDR correction are reported for completeness but not interpreted; following current guidance, we avoid describing sub-threshold results as “trends” or as “approaching significance” (Wasserstein and Lazar, 2016; Amrhein et al., 2019).

## 3 Results

### 3.1 Clinical EEG indices: the MoCA gradient (primary analysis)

The clinical index analyses constitute the primary inferential basis of this paper, as they are the only domain in which results survived Benjamini–Hochberg FDR correction. MoCA continuous gradient (*n* = 19, eleven pre-specified outcomes): 8 of 11 outcomes survived BH correction (adjusted *p* < 0.05; Table 3). The two strongest were EI at Fp2 (ρ = +0.704, *q* = 0.008, *d* = +1.52) and ABR at Fp2 (ρ = −0.687, *q* = 0.008, *d* = −1.49): higher MoCA was associated with greater post-intervention frontal activation (EI increase, ABR decrease). TBR Fp2 (ρ = −0.635, *q* = 0.008), TBHR Fp2 (ρ = −0.589, *q* = 0.018), TAR T4 (ρ = −0.571, *q* = 0.018), TBR F4 (ρ = −0.559, *q* = 0.018), Arousal global (ρ = +0.539, *q* = 0.035), and EI F4 (ρ = +0.532, *q* = 0.035) also survived (Figure 1).

**Table 3:** MoCA gradient on the primary pre-specified clinical EEG indices (*N* = 19).

Index	Electrode	Spearman ρ	Perm *p*	Cohen's *d*	BH *q*	Sig.
EI	Fp2	+0.704	0.002	+1.52	0.008	^**^
ABR	Fp2	−0.687	0.003	−1.49	0.008	^**^
TBR	Fp2	−0.635	0.003	−1.37	0.008	^**^
TBHR	Fp2	−0.589	0.008	−1.27	0.018	^**^
TAR	T4	−0.571	0.010	−1.23	0.018	^**^
TBR	F4	−0.559	0.011	−1.21	0.018	^**^
Arousal	global	+0.539	0.017	+1.16	0.035	^**^
EI	F4	+0.532	0.019	+1.15	0.035	^**^
TBHR	T4	−0.457	0.049	−1.03	0.054	ns
ABR	F4	−0.432	0.065	−0.96	0.068	ns
Valence	global	+0.492	0.0800	+0.36	0.0880	ns

Spearman ρ between baseline MoCA and the pre-to-post change (ΔZ = Post – Pre); permutation p; Cohen's d; Benjamini–Hochberg FDR-adjusted q. Cohen's d is computed as the mean ΔZ divided by the standard deviation of ΔZ across the 19 participants [i.e., *d* = mean(ΔZ)/SD(ΔZ)], using the pre-to-post change as the effect-size denominator; it reflects the magnitude of the average shift relative to inter-individual variability in change, not a between-group comparison. ^**^q < 0.05 (survives FDR correction). Three indices (TBHR at T4, *q* = 0.054; ABR at F4, *q* = 0.068; and Valence, *q* = 0.088) did not survive correction and are shown in the lower rows for completeness only; sub-threshold results are not interpreted (Amrhein et al., 2019; Wasserstein and Lazar, 2016).

**Figure 1:**
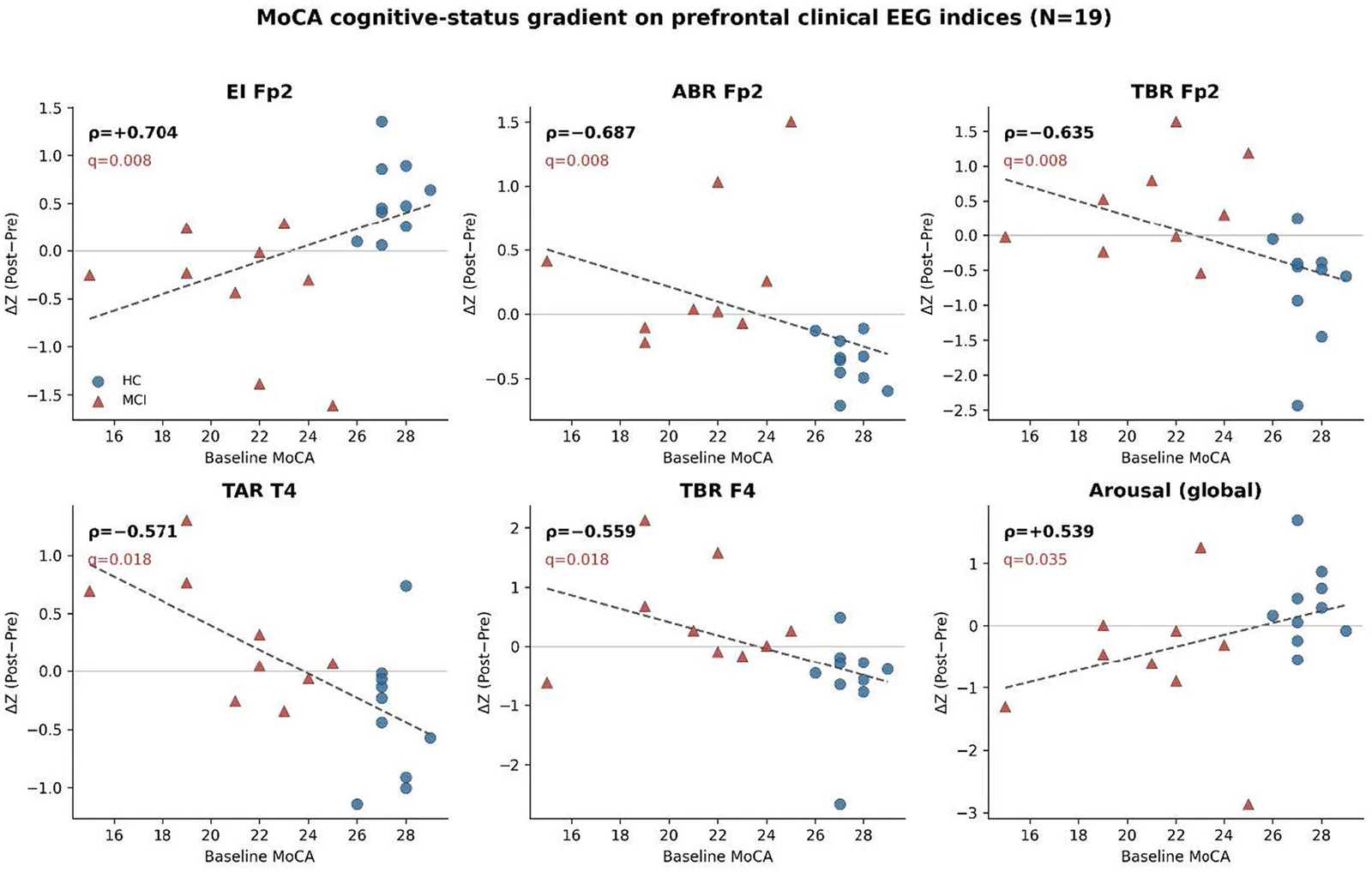
MoCA cognitive gradient on clinical EEG indices (*N* = 19). Scatter plots of baseline MoCA against the pre-to-post change (ΔZ = Post–Pre, Z-score units) for the pre-specified clinical indices that survived Benjamini–Hochberg FDR correction (Engagement Index, Alpha/Beta Ratio and Theta/Beta Ratio at Fp2, and the related prefrontal indices; see Table 3). Red triangles: MCI; blue circles: HC. Higher baseline MoCA is associated with greater post-intervention normalization of prefrontal activation.

Figure 2 illustrates the spatial topology of the between-group contrast, the MoCA gradient across electrodes, and individual Pre → Post trajectories at prefrontal sites—where EI and ABR show qualitatively opposite group directions (see topographic maps in Figure 2, and tendency in Figure 3).

**Figure 2:**
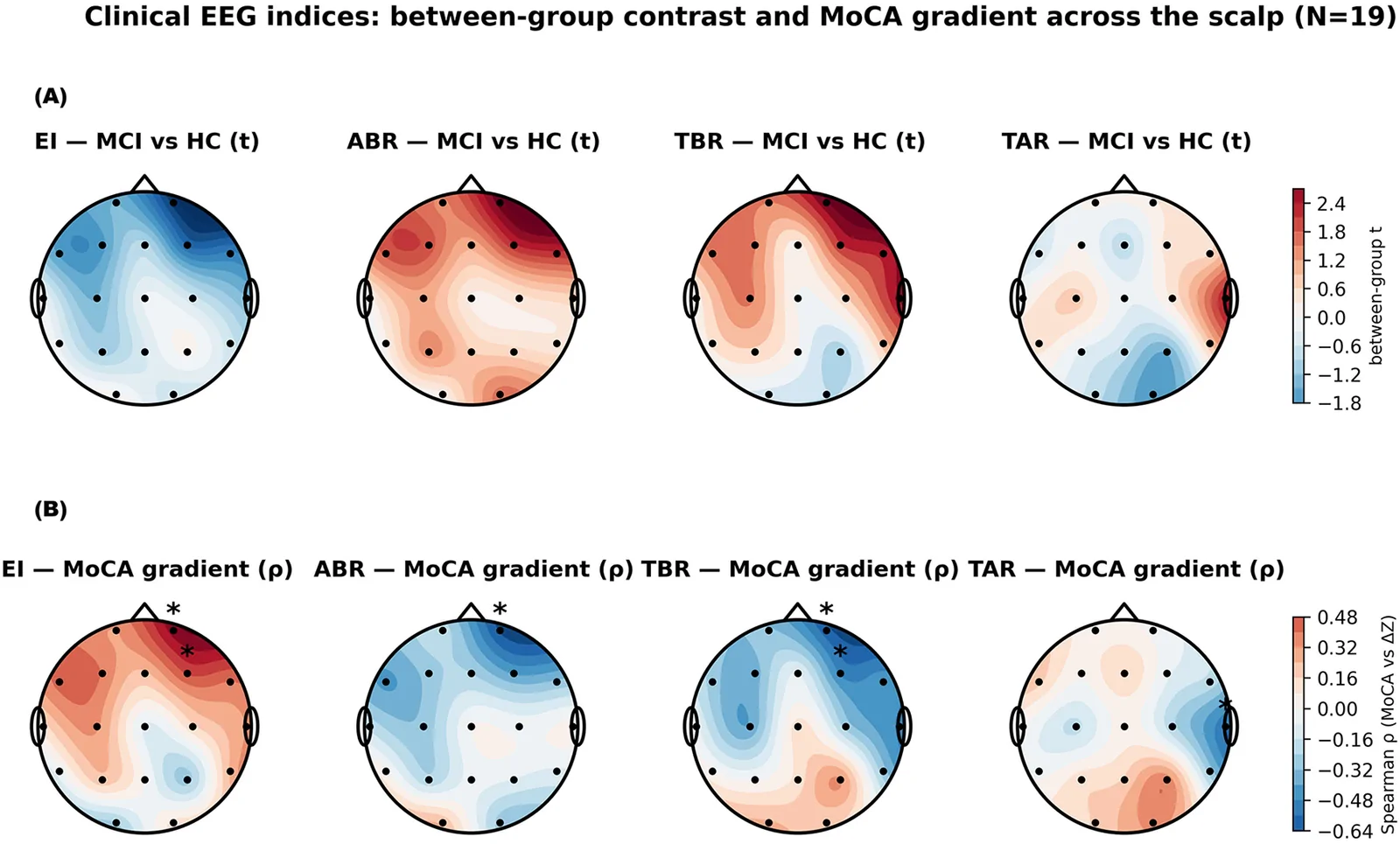
Clinical EEG indices: binary group effect and MoCA gradient. **(A)** Topographic maps of the permutation t-statistic for the between-group contrast (MCI vs. HC) for five clinical indices (EI, ABR, TBR, TAR). Red: MCI > HC; blue: HC > MCI; ⋆ *p* < 0.05; Δ *p* < 0.10. **(B)** Topographic maps of permutation Spearman ρ between baseline MoCA and post-intervention EEG change for the same indices. Orange/red: higher MoCA predicts greater increase (HC direction); green/blue: lower MoCA predicts greater increase (MCI direction).

**Figure 3:**
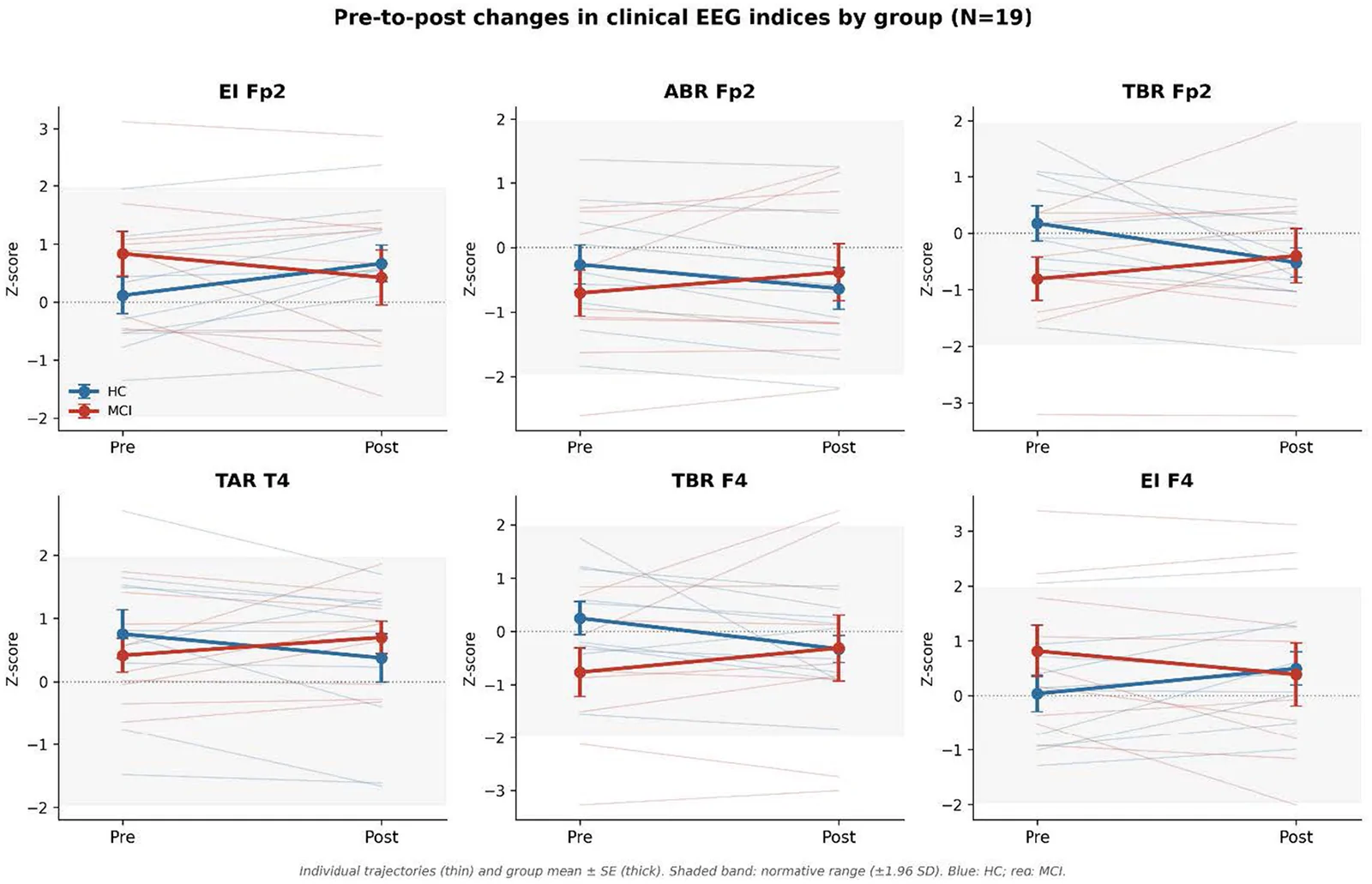
Pre-to-post intervention changes in clinical EEG indices by group (*N* = 19). Thin lines: individual participants. Thick lines with error bars: group mean ± SE. Blue: HC (*n* = 10); red: MCI (*n* = 9). Each panel shows a different index–electrode pair, normalized against the HarMNqEEG reference database (Z-score units; dotted line at *Z* = 0; shaded band: normative range ±1.96 SD). Top row (prefrontal indices, Fp2): EI, engagement index; ABR, alpha/beta ratio; TBR, Theta/beta ratio. Bottom row (temporal/frontotemporal indices): Theta/Alpha Ratio (TAR) at T4, TBR at F4, EI at F4. All findings are exploratory; no within-group change survived BH correction. The qualitatively opposite trajectories at prefrontal sites (e.g., EI and ABR in HC vs. MCI) are consistent with the continuous MoCA gradient reported in Figure 1.

## Movement toward the normative range: index-level summary

Table 4 provides a descriptive illustration—not independent inferential evidence—of the group-level pattern implied by the BH-corrected MoCA gradient. It displays, for each group separately, the mean Z-score before and after the intervention and the mean absolute distance from the normative mean (Mean |Z|), together with the proportion of participants within the normative range (±1.96 SD). None of the within-group changes shown in this table survive Benjamini–Hochberg FDR correction; no *p*-values from this table should be interpreted as inferential results. The table is included solely to show that the directionality visible in Figure 3—MCI participants tending toward the normative mean, HC participants remaining within it—is consistent, at the group level, with the continuous MoCA gradient established in §3.1. Readers interested in the corrected effect sizes and exact *q*-values are referred to Table 3.

**Table 4:** Pre-to-post movement toward the normative range for selected clinical EEG indices (MCI *n* = 9; HC *n* = 10).

Index	Electrode	Group	Pre Z (group mean)	Post Z (group mean)	ΔZ	Mean |Z| Pre	Mean |Z| Post	Δ Mean |Z|	% within ±1.96 SD Pre → Post	*d* (paired)
TBR	F8	**MCI**	−1.64	−0.65	+0.99	1.96	1.72	–**0.24**	**67** ** → 78%**	***d*** **=** +0.74^*^
TBR	F7	**MCI**	−0.96	−0.42	+0.54	1.20	0.94	–**0.27**	**78** ** → 100%**	***d*** = +0.47
EI	F8	**MCI**	+1.63	+0.87	−0.76	1.93	1.51	–**0.41**	78 → 78%	***d*** **=** −0.70^*^
EI	T4	**MCI**	+0.45	+0.06	−0.39	1.11	0.82	–**0.29**	**89** ** → 100%**	***d*** **=** –1.00^**^
EI	T3	**MCI**	+0.27	+0.01	−0.26	0.96	0.65	–**0.31**	100 → 100%	*d* = −0.49
ABR	F8	**MCI**	−1.37	−0.89	+0.48	1.62	1.30	–**0.32**	78 → 78%	*d* = +0.61
ABR	T4	**MCI**	−0.53	−0.27	+0.26	1.12	0.89	–**0.23**	89 → 89%	***d*** **=** +0.67^*^
TAR	P4	**MCI**	+0.67	+0.23	−0.43	1.31	1.13	−0.18	**78** ** → 89%**	***d*** **=** –0.85^**^
Arousal	—	**MCI**	+1.33	+0.74	−0.58	1.46	1.20	–**0.26**	78 → 78%	*d* = −0.52
TBR	F8	**HC**	+0.17	+0.05	−0.12	0.52	0.78	+0.26	100 → 100%	*d* = −0.16
TBR	F7	**HC**	+0.10	−0.13	−0.23	0.55	0.83	+0.28	100 → 100%	***d*** **=** **−0.32**
EI	F8	**HC**	+0.07	+0.28	+0.21	0.60	0.70	+0.11	100 → 90%	*d* = +0.39
Arousal	—	**HC**	+0.13	+0.46	+0.33	0.71	0.72	+0.01	90 → 90%	*d* = +0.52

Pre Z / Post Z: group mean Z-score. ΔZ: Post–Pre. Mean |Z|: mean of individual absolute Z-scores (average distance from population norm, regardless of direction). Δ Mean |Z|: change in mean absolute distance (green = movement toward norm). % within ±1.96 SD: proportion of participants within normative range; green = improvement. d: Cohen's d paired. ^*^p < 0.10; ^**^p < 0.05 (paired t-test, uncorrected; no result survived BH correction). Green rows = MCI; blue rows = HC. ΔZ = Post – Pre of group means. Orange shading in the Δ Mean |Z| column indicates a slight post-intervention increase in distance from the normative mean (movement away from norm). All orange values occur in the HC group, which was already within the normative range at baseline; magnitudes are small (+0.01 to +0.28 Z-score units) and none survived BH correction.

Table 4 does not constitute inferential evidence. The uncorrected paired-test statistics reported in the *d* column are provided to characterize effect magnitude within the MCI subgroup, not to claim significance; with nine observations and no correction applied, these values carry no inferential weight. They are consistent with, and subsumed by, the BH-corrected MoCA gradient in Table 3, which remains the sole inferential result of this paper.

Exploratory correlations between game-learning rates and EEG outcomes did not survive BH correction () and are not interpreted further.

### 3.2 Robustness to educational attainment

Educational attainment differed markedly between the two cognitive-status strata (HC: 13.9 ± 2.8 vs. MCI: 6.9 ± 2.7 years; *t* = 5.58, *p* < 0.001, *d* = 2.56), consistent with the well-documented clustering of low education, physical inactivity, and cognitive decline in older adults from low- and middle-income settings (Cai et al., 2023). Body weight did not differ meaningfully between groups (HC: 63.1 ± 14.0 vs. MCI: 66.6 ± 13.9 kg; *t* = −0.54, *p* = 0.60, *d* = −0.25) and showed no correlation with baseline MoCA (ρ = −0.01, *p* = 0.97), indicating that weight was not a confound in this sample. Education correlated substantially with MoCA at the whole-sample level (*r* = 0.596, *p* = 0.007; 36% shared variance), but this association was driven by the HC/MCI contrast: within each group, the correlation was negligible (HC: *r* = −0.65; MCI: *r* = −0.16). Both variables independently predicted the electrocortical change (e.g., ΔEI Fp2: MoCA ρ = +0.704, education ρ = +0.685), reflecting their shared indexing of the same underlying cognitive-status gradient rather than independent causal pathways. Attempting to disentangle MoCA and education as separate predictors in a partial correlation with *n* = 19 observations and 17 degrees of freedom produces an underpowered model in which no index survives BH correction after adjustment (best partial ρ = +0.50, *p* = 0.033, *q* = 0.187). This statistical limitation is inherent to the sample size and does not alter the primary finding; we report the partial correlation for transparency.

## 4 Discussion

This study asked, as an exploratory question, whether the resting-state electrocortical response to a sensory-motor cognitive intervention in older adults is graded by baseline cognitive status. The single analytical level that provided BH-corrected evidence was the continuous MoCA gradient on pre-specified clinical EEG indices.

### 4.1 The continuous MoCA gradient

The MoCA gradient in clinical indices (8 out of 11 outcomes surviving BH correction; Table 3) defines the primary empirical basis for the graded interpretation. The two anchor findings—EI at Fp2 (ρ = +0.704, *q* = 0.008) and ABR at Fp2 (ρ = −0.687, *q* = 0.008)—represent complementary poles at the same prefrontal electrode: higher MoCA associates with greater frontal activation (EI increase, ABR decrease), lower MoCA with the reverse.

The co-variation of MoCA and educational attainment (*r* = 0.596, driven by the HC/MCI contrast) reflects a well-characterized epidemiological pattern: in older adults from Latin American and low- and middle-income settings, lower education, greater sedentary behavior, and lower cognitive scores cluster as expressions of the same socioeconomic gradient (Cai et al., 2023). Body weight did not co-vary with MoCA (ρ = −0.01), confirming it was not a confound. Because education and MoCA both index the same cognitive-status dimension through different routes, they cannot be cleanly disentangled with *n* = 19; the gradient reflects overall cognitive status rather than any single predictor.

### 4.2 Limitations and future directions

Several limitations must be acknowledged. First, the EEG sample (*n* = 19; 9 MCI, 10 HC) is small and the design is single-arm; all findings are exploratory and hypothesis-generating. Second, the absence of a randomized control group means that regression to the mean (RTM) cannot be excluded as a partial contributor to the observed changes. However, RTM alone is insufficient to account for the primary finding. RTM predicts change proportional to an observation's initial distance from the mean, regardless of any other characteristic of the participant. The BH-corrected MoCA gradient (Table 3) shows instead that the direction and magnitude of post-intervention change co-vary continuously and monotonically with baseline cognitive status—a relationship that RTM has no mechanism to produce. Two participants equally distant from the normative mean at baseline but differing in MoCA would, under RTM, show equivalent regression; the data show systematically divergent trajectories. The prefrontal specificity of this pattern (concentrated at Fp2 and F4 across multiple indices) and its graded dependence on a continuous cognitive variable are both inconsistent with RTM as a complete explanation, though it may contribute to the magnitude of the within-MCI change. Disentangling the RTM component from the intervention effect requires a randomized control group, which motivates the planned RCT.

Third, the HarFEDPNorms normative reference was derived from an independent multinational database of 1,564 healthy adults across 9 countries and 19 sites (Li et al., 2022), entirely distinct from the intervention sample; there is no circularity in the Z-score computation. Fourth, the 51 participants not enrolled from the initial screen of 70 did not meet the pre-established inclusion criteria (independent ambulation, absence of uncontrolled systemic or neurodegenerative disease, stable medication for ≥90 days, and willingness to complete the full 30-session protocol); their exclusion reflects eligibility screening, not selection bias. Fifth, educational attainment co-varied with MoCA (*r* = 0.596, driven by the HC/MCI contrast), consistent with the epidemiological clustering of low education and cognitive decline in this population. With *n* = 19 and 17 degrees of freedom, partial-correlation adjustment for education is underpowered to disentangle the two predictors, and no index survived BH correction after adjustment. This is a known limitation of small pilot studies and motivates the adequately powered RCT. Finally, the Kinestesia software platform is available upon reasonable request to the corresponding author.

## 5 Conclusions

In this small, uncontrolled exploratory sample, the resting-state electrocortical response to Kinestesia was graded by baseline cognitive status: participants with higher baseline MoCA showed greater post-intervention normalization of prefrontal activation indices, most robustly the Engagement Index and Alpha/Beta Ratio at Fp2. This prefrontal engagement effect survived FDR correction and adjustment for educational attainment. The findings are hypothesis-generating and require confirmation in a randomized controlled trial; the HarFEDPNorms pipeline provides a device-independent framework for individualized EEG tracking in such trials.

## Data Availability

The raw data supporting the conclusions of this article will be made available by the authors, without undue reservation.
